# Neural Changes following Behavioral Activation for a Depressed Breast Cancer Patient: A Functional MRI Case Study

**DOI:** 10.1155/2012/152916

**Published:** 2012-05-09

**Authors:** Michael J. Gawrysiak, John P. Carvalho, Baxter P. Rogers, Christopher R. N. Nicholas, John H. Dougherty, Derek R. Hopko

**Affiliations:** ^1^The Department of Psychology, University of Tennessee, Austin Peay Building, Knoxville, TN 37996-0900, USA; ^2^Cole Neuroscience Center, Memory Disorder Clinic, University of Tennessee Medical Center, Knoxville, TN 37920, USA; ^3^Mental Illness Research, Education, and Clinical Center, Philadelphia Veterans Affairs Medical Center, Philadelphia, PA 19104, USA; ^4^Institute of Imaging Science, Vanderbilt University, Nashville, TN 37232, USA

## Abstract

Functional neuroimaging is an innovative but at this stage underutilized method to assess the efficacy of psychotherapy for depression. Functional magnetic resonance imaging (fMRI) was used in this case study to examine changes in brain activity in a depressed breast cancer patient receiving an 8-session Behavioral Activation Treatment for Depression (BATD), based on the work of Hopko and Lejuez (2007). A music listening paradigm was used during fMRI brain scans to assess reward responsiveness at pre- and posttreatment. Following treatment, the patient exhibited attenuated depression and changes in blood oxygenation level dependence (BOLD) response in regions of the prefrontal cortex and the subgenual cingulate cortex. These preliminary findings outline a novel means to assess psychotherapy efficacy and suggest that BATD elicits functional brain changes in areas implicated in the pathophysiology of depression. Further research is necessary to explore neurobiological mechanisms of change in BATD, particularly the potential mediating effects of reward responsiveness and associated brain functioning.

## 1. Introduction

Investigating neurobiological processes associated with depression treatment is a burgeoning area of translational research. Major depression is conceptualized as a systems level disorder affecting cortical, subcortical, and limbic regions [1–4]. Much of the aberrant functional brain activity in these areas tends to normalize with symptom remission [5–7]. Understanding the putative mechanisms of change facilitated by psychosocial treatments for depression may enhance our understanding of the pathophysiology of the disorder, lead to treatment refinement and development, and facilitate improved patient-treatment matching [8]. Investigation of neurobiological processes also can provide valuable data and hypotheses pertaining to the etiology and maintenance of depression in difficult-to-treat patient samples, such as those with early onset chronic depression [9, 10]. For example, data suggest that this patient population may present with deficits in formal operational thought, mentalization abilities, and an underdeveloped theory of mind [10–13].

Early pioneering studies evaluating therapy outcome and brain activity examined Interpersonal Psychotherapy (IPT; [5, 14], Cognitive Behavioral Therapy (CBT; [15–17]), and Behavioral Activation Treatment for Depression (BATD; [18]), demonstrating that positive treatment outcome is associated with changes in brain regions implicated in the pathophysiology of depression. Such brain changes are thought to reflect improved problem-solving, reductions in negative effect, decreased rumination, and improved self-perception [19–22]. While these findings are salient to understanding the pathophysiology of depression and the role of psychotherapy in modulating aberrant brain activations, assumptions made about functional brain changes are primarily based on resting state brain scans. Utilizing functional tasks during scanning has been encouraged as it more clearly delineates neurobiological components of depression and whether therapy treatments affect theoretically relevant brain regions [23]. Only two studies [15, 18] used functional tasks during scanning that directly translate to clinical practice and more systematically assess activations associated with behavioral models of depression [19, 20]. 

Investigating neurobiological networks of reward is warranted given the relevance of behavioral inhibition, withdrawal, avoidance, and limited behavioral activation among depressed individuals [21–23]. Indeed, the functional brain activity of depressed individuals has been observed to exhibit decreased activation in mesolimbic regions when exposed to smiling faces or pleasant autobiographical narratives [24–26] and to have differential responses in regions implicated in processing other rewarding stimuli such as food, sex, and drugs [27–31]. Although an examination of the neurobiological activity associated with reward responsiveness could inform pathophysiological and behavioral models of depression, to date only one study has examined interrelations among depression, diminished reward response, functional brain activity, and changes following psychotherapy [13]. This study employed functional magnetic resonance imaging (fMRI) to assess Blood Oxygenation Level Dependence (BOLD) response in patients during a reward responsiveness, Wheel-of-Fortune task [13]. Seventy-five percent of depressed patients treated with BATD were treatment responders, and, relative to changes in brain function in a matched nondepressed group, BATD resulted in functional changes in structures that mediate response to rewards, including the paracingulate gyrus during reward selection, the right caudate nucleus (the dorsal striatum), during reward anticipation, and the paracingulate and orbital frontal gyri during reward feedback. The neuroimaging data from this study [13] are relevant to both depression and the purported mechanism of change of the treatment, which in BATD is increased exposure to rewarding stimuli [19]. 

Models of depression highlight decreased behavior activation and minimized exposure to reward and environmental reinforcement as primary causal factors in the onset and maintenance of depression [20, 32, 33]. BATD targets structured increases in overt behaviors with the purpose of increasing exposure to reinforcing environmental contingencies and eliciting corresponding improvement in thoughts, mood, and quality of life [19]. Three meta-analyses support behavioral activation interventions as efficacious and empirically validated treatments for depression [34–37]. Particularly relevant to the current study, BATD also has been effective with depressed cancer patients [38–40]. 

Given the efficacy of behavioral activation in treating depression via increased reward exposure, this study evaluated whether BATD corresponded to predictable changes in functional brain activity. To examine this question, a novel reward responsiveness paradigm (pleasurable music listening) [36, 41] was used to explore regional brain activations in a depressed breast cancer patient receiving BATD. We first posited that music listening would be an appropriate fMRI paradigm to evaluate neurobiological reward responsiveness. Following this, we hypothesized that exposure to preferred music passages at pre- and posttreatment would elicit increased activation in cortical and subcortical brain regions involved in reward. Specifically, we expected so see increased activations in the nucleus accumbens, ventral striatum, medial orbital (moPFC), and dorsolateral prefrontal cortex (dlPFC). Secondarily, we hypothesized reduced activity in the globus pallidus and subgenual cingulated cortex. We anticipated that these regional changes would correspond with reductions on self-reportmeasures of depression and behavioral inhibition and an increase in environmental reward and behavioral activation. 

## 2. Case Presentation

The female patient was recruited from an ongoing study at the University of Tennessee Medical Center's Cancer Institute that was a randomized clinical trial examining the efficacy of BATD and Problem-Solving Therapy for depressed breast cancer patients [42]. Based on the Anxiety Disorder Interview for DSM-IV that comprehensively assesses anxiety and mood disorders (ADIS-IV: [43]), she had a primary diagnosis of major depressive disorder, single episode (296.22) and secondary diagnosis of generalized anxiety disorder (300.02) [44]. Relevant for fMRI procedures, she had no history of spinal or brain cancer, right hand dominance as indicated by the Edinburgh Handedness Inventory [45], and no surgical metal implants. Before and after treatment, she completed the Behavioral Inhibition and Activation Scale (BIS/BAS: [46]) to measure motivation and avoidance, the Beck Depression Inventory-II (BDI-II: [47]) to assess depression severity, and the Environmental Reward Observation Scale (EROS: [48]) to assess environmental reward. The BDI-II and EROS were completed after each therapy session. An advanced graduate student in clinical psychology completed the Hamilton Rating Scale for Depression (HRSD: [49]) at pre- and posttreatment. 

The patient in this study was 64 years old, right-handed, married, and Caucasian, with two years of graduate level education in Liberal Arts. She was diagnosed with breast cancer four months prior to her pretreatment evaluation. She received cancer treatment in the form of a lumpectomy, one month following her diagnosis, and chemotherapy that began one month prior to study enrollment that persisted through the course of psychotherapy. Her medication regimen was consistent throughout therapy and was limited to allergy, migraine, and sleep prescriptions. Prior to treatment and throughout the course of BATD, the patient was not medicated with antidepressant or antianxiety medications. She reported no prior history of psychiatric problems other than depression and anxiety that emerged 6 months prior to her cancer diagnosis due to psychosocial stressors (i.e., death of family pet, marital problems, and job dissatisfaction). Her depression significantly exacerbated upon her breast cancer diagnosis and manifested as sleep disturbances, feelings of guilt, worthlessness, and low self-esteem. Her generalized anxiety manifested as restlessness, fatigue, difficulty concentrating, irritability, muscle tension, and insomnia. 

The patient was treated with an 8-session BATD protocol that consisted of sessions approximately 1 hour in duration [50]. Initial sessions consisted of assessing the function of her depressed behavior, efforts to weaken access to positive and negative reinforcement for depressed behavior, and introduction of the treatment rationale. A systematic behavioral activation approach was then initiated to increase the frequency and subsequent reinforcement of healthy behaviors. The patient began with a weekly self-monitoring exercise that served as a baseline assessment of daily activities, oriented her to the quality and quantity of her activities, and generated ideas about activities to target during treatment. In session three, the patient engaged in the life areas and value assessment (LAVA) in which ideographic life values were identified and behavioral goals were established within major life areas: family, peer, and intimate relationships, daily responsibilities, education, employment, hobbies and recreational activities, physical/health issues, spirituality, and anxiety-eliciting situations. Based on the LAVA assessment, 15 overt behaviors were identified (e.g., spending more time with her grandchildren, increasing exercise, gardening, participation in church activities) that would increase environmental reward and response-contingent positive reinforcement. Subsequent treatment sessions focused on progressively increasing engagement in rewarding activities and monitoring progress. All sessions were provided on an outpatient basis at the University of Tennessee Medical Center Cancer Institute. An advanced male clinical psychology doctoral student with extensive training in BATD conducted the psychotherapy. 

### 2.1. Reward Responsiveness Task Design

The 30-minute music listening reward responsiveness paradigm was adapted from previous neuroimaging studies on music listening, reward, and depression [36, 41]. It involved listening to two music tracks, each of which was 7.5 minutes long. Each music track alternated between 50-second segments of silence, preferred, and neutral passages, proceeding through the songs in 50-second intervals. The second track duplicated the first with exception of reversed order of preferred and silence. Selection of preferred and neutral music passages [36] was done prior to the day of the scan by having the patient listen and rate instrumental music passages. Rankings were obtained in intervals of 20 on a likert scale ranging from −100 (disliked completely) to 0 (neither liked nor disliked) to +100 (liked completely). Rankings were considered neutral if rated between −40 and +40 and preferred if rated 60 or higher. The neutral music passage served as the control condition for brain activity associated with a nonrewarding stimulus. The patient was given no instructions during scanning other than to stay focused and remain still. 

### 2.2. Functional MRI Statistical Analysis

The patient was scanned one week prior to BATD and one week following therapy. Imaging was performed on a 1.5-T Siemens MRI scanner at the University of Tennessee, Department of Radiology. Data processing and analyses utilized Statistical Parametric Mapping (SPM8) methods (Wellcome Department of Cognitive Neurology, London, UK). Pre- and post-treatment scans were included in a single massively univariate general linear model. Regressors were included for each condition (neutral or preferred; music or silence) to indicate music listening for each run of each session. These images consist of appropriate boxcar functions convolved with a canonical hemodynamic response shape. Within each session the contrast of BOLD signal during the preferred music relative to the neutral music was used as a measure of brain response to reward. Contrasts examining BOLD signal during music relative to silence were also assessed as an indirect measure of reward. These measures were compared between sessions using appropriate contrasts to examine the effect of treatment. 

The SPM *T* maps of the contrast of interest were thresholded at *T* = 2.58 (voxelwise *P* < 0.005). The statistical significance of the resulting clusters was calculated using the approach of random field theory [51, 52]. With knowledge of the search volume (number of total voxels) and the smoothness of the *T* map images, this methodology allows for calculating the probability of a suprathreshold cluster of a particular size occurring by chance. To improve sensitivity, this statistical analysis was limited to an a priori region of interest using small volume correction methodology [53]. By limiting the volume searched to only part of the brain, the statistical corrections applied can be less stringent, allowing better sensitivity to small changes at the cost of missing activations outside the a priori region. The regions of interest included brain areas related to reward responsiveness and depression treatment outcome and were defined as the union of the frontal pole, orbital frontal cortex, caudate, putamen, accumbens, subcallosal, medial frontal, middle, and superior frontal gyrus, anterior and posterior cingulate, and the paracingulate, from the Harvard-Oxford probabilistic atlas [54–57] implemented in FSLView v3.0 (http://www.fmrib.ox.ac.uk/fsl/fslview/index.html). Clusters that showed significant responses at the uncorrected cluster-level *P*-value of 0.05 were tabulated and reported along with *P*-values corrected for multiple comparisons at the whole brain level. 

### 2.3. Treatment Outcome

**Figure 1:**
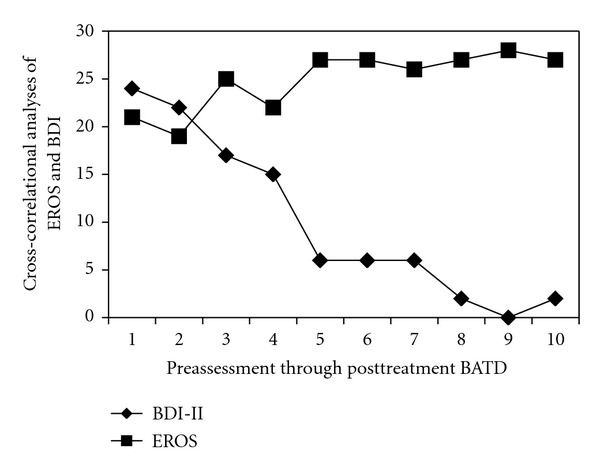
Cross-correlational analyses (CCA) of EROS and BDI-II from preassessment, through each therapy session, and following completion of BATD. CCA statistics showed that BDI-II and EROS scores were statistically significant at lag 0 (*r* = −0.92, *P* = 0.000). BDI-II and EROS points reflect measures completed at preassessment, during each of the 8 therapy sessions, and following BATD completion.

Based on reliable change indices [58] established through treatment outcome research with depressed breast cancer patients [42], the patient exhibited clinically relevant symptom changes at post-treatment (see Table 1) and was considered to be in full remission of her depression [42]. To further assess changes observed in measures of depression and environmental reward, a cross-correlation analyses (CCA) was conducted using the Simulation Modeling Analysis software (SMA; [53]) to determine the extent to which changes in weekly session measures were related throughout therapy. CCA determines the degree that two variables are related to each other at a specified interval. For the patient, the two measures most highly correlated at lag 0, indicating that BDI-II scores were most strongly related to EROS scores on a session-by-session basis. CCA statistics showed that BDI-II and EROS scores were statistically significant at lag 0 (*r* = −0.92, *P* = 0.000; see Figure 1). 

### 2.4. Functional MRI Data

**Figure 2:**
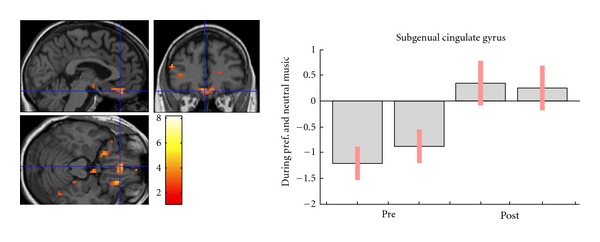
*T*-Maps and plot denoting BOLD response. Response to music, agnostic to valence (preferred and neutral versus silence), changed after treatment in the subgenual cingulate (−3 35 −20). BOLD response was deactivated for music conditions, relative silence, before treatment. At posttreatment, this region showed little distinction between music and silence, evidencing an overall reduction in activity. Neurological convention (right on right) is used, and coordinates are in Montreal Neurological Institute space. Statistical map thresholded at *P* < 0.005. Bar plots show the BOLD responses in the marked regions, and red bars are 90% confidence intervals.

We assessed the effect of treatment on two types of BOLD response: preferred versus neutral music (time-by-valence interaction) and all music versus silence (time-by-music interaction). There was a significant effect of treatment on the BOLD response to music versus silence (time-by-music interaction, *P* < 0.05 corrected) in only one region, the subgenual cingulate (see Table 2). At pretreatment, the patient exhibited strong reductions in subgenual cingulate BOLD response during music relative to silence. At posttreatment, subgenual BOLD response was just slightly higher during music relative to silence (see Figure 2). There were no effects of treatment on the BOLD response to preferred versus neutral music (time-by-valence interactions) at the *P* < 0.05 corrected level. However, several regions evidenced changes at *P* < 0.05 (uncorrected) that were largely consistent with apriori predictions (see Table 2 for complete list of contrasts, regions of interest, and corresponding statistics). These regional changes included increased activations in the moPFC and dlPFC following treatment. There were no changes, at corrected or uncorrected thresholds, for any contrasts examining time-by-music valence or time-by-music interactions in any of the subcortical regions (i.e., nucleus accumbens, ventral striatum, and globus pallidus) hypothesized to be affected by treatment. 

## 3. Discussion

This case study explored changes in depression severity and functional brain activity following 8 sessions of BATD for a depressed breast cancer patient. The patient responded favorably to treatment, as reflected by clinically significant changes on clinician and self-report measures of depression, and she was in full depression remission at post-treatment. A direct inverse relationship between self-reported depression and environmental reward was evident via cross-correlational analyses, with depression attenuation associated with increased environmental rewards. Accordingly, these data are generally supportive of behavioral models of depression where increased environmental reward is proposed to mediate the relationship between increased overt activity and depression reduction [25, 59, 60]. 

Our first hypothesis was only partially supported, as the music listening fMRI paradigm did not elicit activity in subcortical regions implicated in reward responsiveness and depression. To speculate on this finding, either the rewarding music paradigm was insufficient to elicit neural underpinnings of reward responsiveness or the case study design restricted the power necessary to observe these changes. The latter explanation is suspected as similar scanner paradigms have demonstrated efficacy in eliciting reward responsiveness neural activity [36]. A third possibility is that BATD does not exert changes via a direct effect on subcortical neural circuits of reward and that these regions are affected as a secondary consequence of frontal cortical regions being engaged during psychotherapy. 

Statistically significant changes emerged in the subgenual cingulate for the contrast examining music and silence. At pretreatment, the patient exhibited reduced BOLD response in this region during the music conditions relative to silence. Following treatment, BOLD response evidenced a very slight activation during music relative to silence, though the difference between music and silence was marginally distinguishable. This implies an elevated pretreatment BOLD response during silence compared to music, which is an interesting and highly relevant finding given that elevated activity within the subgenual cingulate is considered a hallmark for neurobiological models of depression [6–8, 61], distinguishes healthy from depressed individuals during resting-state scans, and plays a crucial role in emotion processing and the pathophysiology of mood disorders [7, 62]. Moreover, the subgenual cingulate tends to decline in activity in depressed patients responsive to treatment [6, 63]. Interestingly, this region attenuated in overall activity following BATD, evidencing a deactivation during the silent condition, relative to music, exhibiting little distinction between music and silence exposure. 

Several results observed at the more lenient threshold *P* < 0.05 (uncorrected) are noteworthy as they supported a priori predictions. These BOLD changes are relevant to treatment and the pathophysiology of depression [4, 8]. Following treatment, BOLD response in the right-sided dlPFC and moPFC increased in activation during preferred music. Similar findings were observed with music and silence for increased BOLD response in the left dlPFC and right moPFC following treatment. These changes may plausibly reflect decreased depressive rumination, increased utilization of cognitive resources, or increased capacity to experience reward during more pleasurable experiences as such phenomena have been associated with dlPFC and moPFC activity [36, 64–66]. However, future studies with comparable methodology and larger sample sizes are needed to replicate these results. 

Several study limitations are noteworthy. First, the case study design and lack of a control group limit the degree to which findings can be generalized to the population. Second, treatment was not independently evaluated to assess therapist competence or treatment adherence. Third, it is not clear to what extent the consistent regimen of allergy and sleep medication or medical treatment constituted an artifact for the patient's depression, treatments, or results of brain scans. Despite these limitations, several findings are noteworthy and contribute to translational research on depression and neurobiological processes. First, while the music paradigm did not effectively elicit subcortical activity associated with reward responsiveness, it did elicit cortical activity implicated in reward, affect regulation, and executive function, which warrants its use in future studies. Second, this is one of only two studies that have identified functional brain changes when BATD is associated with positive treatment outcome. Moreover, few investigations of functional brain activity, following treatment for depression, have examined changes in response to a scanner paradigm as included in this design. Future research examining BATD and the pathophysiology of depression would benefit from employing a wider variety of scanner paradigms to more broadly assess how BATD targets functionally aberrant brain regions. Second, the shared and unique neurological effects of BATD relative to other empirically validated treatments for depression require investigation. Third, examining the dose-response effects (i.e., number of treatment sessions) of BATD on neurological outcomes would be intriguing. Finally, fMRI studies should include larger and heterogeneous patient samples to assess psychological, demographic, and medical variables that may impact pre-post treatment neurological changes. Such data could begin to address how different interventions uniquely or similarly target brain regions implicated in depression, might lead to guiding treatment selection based on different brain signatures or patient characteristics, and might lead to developing treatments that selectively target prominent features of depression. These questions are worthy of scientific inquiry as strides are made to increase understanding of neurobiological processes in depression and improved quality of care for depressed patients.

## Figures and Tables

**Table 1:** Symptom assessment at pre- and posttreatment.

Measures	BATD	
	Pre	Post
BDI-II	24	2
EROS	21	27
HRSD	26	0
BIS	9	10
BAS-Drive	9	10
BAS-Fun	7	7
BAS-Reward Response	7	7

BATD: behavioral activation treatment for depression; BDI: beck depression inventory; EROS: environmental reward observation scale; HRSD: hamilton rating scale for depression; BIS: behavioral inhibition scale; BAS: behavioral activation scale. All scores listed are raw scores.

**Table 2:** BOLD response between pre- and post-BATD.

Contrast	Side	MNI			Size	*P* (cor)	*P* (unc.)	*T*-Value
Region		*x*	*y*	*z*				
*Pre > Post (Pref. > Neu.) *								
Middle frontal gyrus/dlPFC	R	42	17	31	35	0.219	0.016*	4.48
Inferior frontal gyrus/moPFC	L	−27	35	−8	38	0.178	0.013*	4.09
Inferior frontal gyrus/moPFC	R	24	32	−20	18	0.663	0.070	4.23
*Post > Pre (Music > Silence)*								
Subgenual cingulate		−3	35	−20	79	0.012**	0.001*	4.73
Inferior frontal gyrus/dlPFC	L	−51	38	16	36	0.204	0.015*	4.37
Middle frontal gyrus/moPFC	R	21	32	−20	27	0.379	0.031*	4.73
*Pre > Post (Music > Silence)*								
Middle frontal gyrus	L	−36	44	19	46	0.102	0.007*	4.32

MNI: montreal neurological institute coordinates. Size: the number of voxels within a given activation cluster. *T*-Value: Peak *T*-Value activation within that cluster. Cluster-defining threshold was a voxel-level *P* < 0.005, and *P*-values reflect cluster-level corrected and cluster-level uncorrected. **P < 0.05 (corrected). *P < 0.05 (uncorrected).
